# *In vivo* Characterization of Biochemical Variants of Amyloid-β in Subjects with Idiopathic Normal Pressure Hydrocephalus and Alzheimer’s Disease Neuropathological Change

**DOI:** 10.3233/JAD-201469

**Published:** 2021-04-06

**Authors:** Sylwia Libard, Jochen Walter, Irina Alafuzoff

**Affiliations:** a Department of Immunology, Genetics and Pathology, Uppsala University, Uppsala, Sweden; b Department of Pathology, Uppsala University Hospital, Uppsala, Sweden; c Department of Neurology, University of Bonn, Bonn, Germany

**Keywords:** Alzheimer’s disease neuropathologic change, amyloid-β, biochemical variants, idiopathic normal pressure hydrocephalus

## Abstract

**Background:**

Stepwise occurrence of biochemically modified amyloid-β (Aβ) in the brain of subjects with Alzheimer’s disease (AD) has been suggested to be of significance for cognitive impairment. Our previous reports have shown that Aβ is observed in 63% of all subjects with idiopathic normal pressure hydrocephalus (iNPH) suggesting that the majority of iNPH subjects with Aβ are indeed also suffering from AD.

**Objective:**

We assessed the occurrence of biochemically modified Aβ variants, *in vivo,* in subjects with iNPH and in a cohort of postmortem brain samples from patients with dementia.

**Methods:**

We assessed Aβ proteins in 127 diagnostic brain biopsies obtained from subjects with iNPH and in a cohort of subjects with dementia by means of immunohistochemistry.

**Results:**

The pyroglutamylated Aβ (pyAβ) precedes the aggregation of phosphorylated Aβ (pAβ) during the AD neuropathological change progression; moreover, these modified variants of Aβ correlate with hyperphosphorylated tau in the frontal cortical area of human brain. Our results confirm the existence of the suggested biochemical stages of Aβ aggregation that might be of significance for neurodegeneration leading to cognitive impairment.

**Conclusion:**

The observation that both pyAβ and pAβ are seen *in vivo* in iNPH subjects is intriguing. It has been reported that most of the iNPH subjects with Aβ in the brain biopsy indeed develop AD with time. Based on our current and previous results, it is clinically merited to obtain a diagnostic biopsy from a subject with iNPH. When Aβ is observed in the biopsy, the biochemical characterization is of interest.

## INTRODUCTION

Extracellular amyloid-β (Aβ) aggregates and intraneuronal accumulation of hyperphosphorylated tau (HP*τ*), i.e., Alzheimer’s disease neuropathological change (ADNC), are the hallmark lesions of AD [1, 2]. During the progression of the disease, the proteins are seen in different brain regions following predisposed neuroanatomical regions, causing a progressive neurodegeneration, leading to cognitive impairment [2–5]. During the last two decades, several groups have been able to detect Aβ and HP*τ*, by applying immunohistochemical (IHC) methods in stereotactic, cortical, brain biopsies from patients surgically treated for idiopathic normal pressure hydrocephalus (iNPH) [6–10].

iNPH is a neurological disease in elderly, caused by impaired cerebrospinal fluid (CSF) circulation, which causes progressive hydrocephalus and presents with gait disturbance, urinary incontinence, and cognitive impairment [11, 12]. Currently, the only treatment strategy implemented in patients with iNPH is a surgical ventriculo-peritoneal shunt (VPS) insertion, which can alleviate the symptoms by normalizing the CSF flow [11, 12]. During the VPS surgery, one tip of the shunt is placed into the frontal horn of the right ventricle and the other in the peritoneal cavity. Both catheters are connected to a shunt valve controlling the CSF pressure [11, 12]. While preparing the shunt channel, some neurosurgeons have taken a diagnostic brain biopsy from the area of the shunt channel, i.e., right frontal lobe [6, 9, 10, 13]. Detection of ADNC by applying IHC method in these samples, from the frontal cortex, suggests at least low to intermediate level of ADNC in the brain, according to international consensus criteria [14, 15]. The presence of ADNC, associated with poor shunt response, has been reported to indicate poorer prognosis and eventual progression to full blown AD [9, 10, 16–18]. When a postmortem (PM) analysis has been carried out on brains obtained from subjects with a clinical diagnosis of iNPH, it has been observed that a substantial number of subjects display varying degrees of ADNC [13, 19, 20]. The early signs of neurodegeneration, especially synaptic loss, seen in AD, were also observed in a cohort of subjects with iNPH [8, 21]. All of the above implies that iNPH seems to be a reliable model of AD.

Aβ, one of the hallmark proteins of AD, is a result of a two-step enzymatic cleavage of the amyloid-β protein precursor, a cell membrane protein, by β- and *γ*-secretase into an Aβ peptide, which is prone to forming extracellular aggregates within the grey matter of the brain [1, 22]. The Aβ is first seen in the neocortex and thereafter progresses through defined neuroanatomical regions, affecting the brainstem and cerebellum at the end stage [3, 23]. The Aβ aggregates can be detected in brain tissue of young subjects, years before the symptom onset of AD and in subjects that never develop a dementing illness [24–26]. The most commonly used antibodies for detection of Aβ are clones Aβ^6F/3D^ and Aβ^4G8^. In addition to Aβ neuroanatomical progression, the protein deposits undergo biochemical changes [22, 27–29]. These biochemical changes primarily affect the N-terminal sequence of the protein [22, 29]. The modified, N-terminal truncated Aβ variants are more toxic and more prone to aggregate when compared with the unmodified Aβ. The pyroglutamylation of the N-terminus produces a py Aβ N3pE variant (pyAβ), which is detected in Aβ aggregates of AD, demonstrating increased tendency to aggregate, increased neurotoxicity, and weak solubility. The most studied phosphorylation site in Aβ is at serine residue 8, producing a phosphorylated Aβ 1E4E11 (pAβ) variant. When this neurotoxic pAβ variant is detected in AD, it increases the formation of Aβ oligomers that form the cores of fibrillization [22]. Previous studies have suggested that the biochemical changes in the composition of Aβ deposits, as mentioned above, occur in a hierarchical manner. Initially, at the pre-clinical AD, stage 1, primarily unmodified Aβ variants are seen in the Aβ aggregates. Parallel to the progress of the pathology, stage 2, the pyAβ variants are detected. At the end stage, when cognitive failure is obvious, the pAβ variants are noted [27, 28]. Furthermore, the pyAβ variant seems to be associated with increased extent of HP*τ* pathology in the human brain [30].

The aim of this study was to assess the presence of different biochemical variants of Aβ and HP*τ*, not only as previously described in PM brain but also in surgical brain biopsy samples obtained from subjects with iNPH treated with VPS. This approach gives us a unique opportunity to study protein expression at an early stage *in vivo*. Furthermore, it allows us to avoid tissue alterations associated with agonal state, PM delay and particularly alterations related to issues such as fixation.

## MATERIAL AND METHODS

### Ethical statement

Regarding biopsies, the study has been approved by the regional Ethical Committee of Uppsala, Sweden #2013/176, updated 2016. The subjects studied here have given their informed consent for the use of the diagnostic tissue for scientific purposes. The use of PM tissue was approved by the regional Ethical Committee of Uppsala, Sweden, #2011/286, updated 2015.

### Study subjects

#### iNPH subjects

All diagnostic brain biopsies from iNPH patients, obtained during curative VPS insertion at Uppsala University Hospital (UUH), during 2010–2018, were identified in the database, Laboratory Investigation System, of the Surgical Pathology Department at UUH. In total, 448 samples were identified. One of the selection criteria was notable Aβ pathology in the biopsy; based on the data in the files, 142 fulfilled this requirement. The second selection criterion was age, i.e., over 70 years at biopsy, and 130 subjects were within the range of 70 to 88 years when biopsied. All the diagnostic slides of this cohort were retrieved from the archives and reassessed. Two cases displayed only sparse Aβ pathology and were excluded from the cohort. Additionally, one subject was biopsied twice; so, the second biopsy from the same subject was excluded from the cohort. Thus, brain biopsy specimens from 127 subjects fulfilled the selection criteria and were included in the study cohort.

#### Biopsy samples

The brain tissue specimen was obtained from the right frontal lobe, within the area of the superior- and medial- right frontal gyri, during the surgical VPS insertion, as previously described [6, 7, 11]. The samples were fixed in 10% neutral buffered formalin (4% formaldehyde), at room temperature for 24 h and then processed into paraffin blocks (Histowax from Histolab Products). The blocks were sectioned, into 4-μm thick sections, which were put on the Super Frost slides for Hematoxylin-Eosin (HE) staining and Super Frost Plus slides for IHC stainings.

### Reference material

A tissue micro array (TMA) block was constructed, including core samples measuring 2 mm in diameter obtained from the amygdaloid body from 26 PM brains. These brains had undergone a standardized neuropathological examination at UUH during 2011–2013 [31]. Each subject was represented by two cores. At the neuropathological investigation, 20 of the subjects displayed various levels of ADNC pathology, whereas six displayed pure Primary Age Related Tauopathy (PART) and thus excluded. The block was sectioned into 4-μm thick sections, which were put on the Super Frost slides for HE staining and Super Frost Plus slides for IHC stainings. Two core samples, from the same subject, were damaged and thus excluded from the final analysis, i.e., assessable material included cores from 19 subjects.

### Immunohistochemistry

The IHC stainings were performed using automatic platform. The antibodies used and the pre-treatments applied are summarized in Table 1. Two of the antibodies, the pAβ^1E4E11^ and the Aβ^7H3D6^, a variant that recognize Aβ with unmodified N-terminus (umAβ), were both generated as previously described [32]. For these two antibodies, additional treatments were implemented following systematic testing in order to reach optimal results. The stainings were performed using Dako Autostainer Plus (DakoCytomation, Glostrup, Denmark) with the Dako EnVision Flex detection system (DakoCytomation), according to the manufacturer’s instructions.

**Table 1:** Immunohistochemical stains

Antibody	Clone	Company/code	Dilution	Pre-treatment	Additional strategy
Aβ aa17–24	4G8	Biolegend/800703	1:4000	FA 5 min
Aβ aa8–17	6F/3D	Dako-Agilent/M0872	1:50	FA 5 min
pyAβ N3pE	polyclonal	Tecan/JP18591	1:50	FA 5 min
pAβ S8 PM	1E4E11	“In house” [32]	1:500	FA 3 min	a
pAβ S8 PX	1E4E11	“In house” [32]	1:500	FA 3 min	b
umAβ	7H3D6	“In house” [32]	1:1000	FA 5 min	c
Hyperphosphorylated (Ser202/Thr205) *τ* (TAU8)	PHF-TAU-AT8	Fisher Scientific-Invitrogen/MN1020	1:1000

Aβ, amyloid-β; py, pyroglutamylated; p, phosphorylated; S8, serine residue 8; um, unmodified; PM, post-mortem; PX, biopsy sample; FA, formic acid 100%. ^a^Primary antibody (Ab) was incubated overnight at 4°C. For detection, a mouse linker was applied for 20 min, followed by Dako Envision Flex Kit. ^b^Incubation with primary Ab 2 x 30 min, followed by mouse linker for 20 min, followed by Dako Envision Flex Kit and 2 x 5 min of 3,3’-Diaminobenzidine/DAB (Horseradish Peroxidase included in Dako Envision Flex Kit). ^c^A rabbit anti-rat Ab (ab6703) was applied for 30 min before applying the Dako Envision Flex Kit.

### Assessment of the samples

All samples were assessed using light microscopy (Olympus BX45) at ×20 to ×400 magnification. The stained sections were then scanned into digital slides with Aperio AT2 (Leica Biosystems, Inc) in 20× magnification, into ScanScope virtual slide (svs) format.

**Fig. 1:**
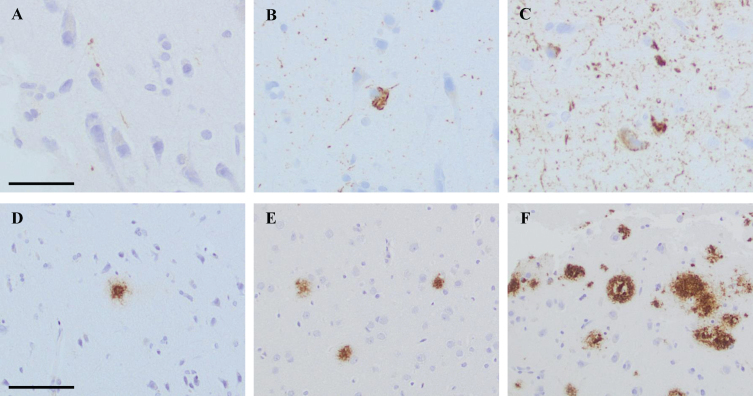
Photos of brain biopsy samples from right frontal cortex, stained by means of immunohistochemistry (IHC). In A-C, IHC outcome at different levels of pathology when applying antibody (Ab) towards hyperphosphorylated tau (HP*τ*, AT8). In A grade 1 = low level of pathology, in B grade 2 = moderate level of pathology and in C grade 3 = high level of pathology. In D, IHC outcome at grade 1 = low level of pathology when applying Ab towards pyroglutamylated Aβ N3pE. In E and F, IHC outcome at different levels of pathology when applying Ab toward Aβ^6F/3D^. In E, grade 2 = moderate level of pathology and in F grade 3 = high level of pathology. In A-C, bar 50μm. In D-F bar 100μm.

The extent of the pathology within each sample was assessed, and the alteration was graded as follows: 0 = no pathology, 1 = low level of pathology, 2 = moderate level of pathology, and 3 = high level of pathology. Low level of HP*τ* pathology was assigned when a single–a few HP*τ* reactive granules and threads were detected. In the moderate level of HP*τ* pathology, scattered granules and threads were seen as well as a few tangles. High level of HP*τ* pathology was assigned when abundant amounts of neurites and several tangles were seen in the sample (Fig. 1). When assessing the Aβ pathology, low level was assigned when a single–couple aggregates were seen in a tissue sample. Moderate level of pathology was assigned when scattered Aβ aggregates were seen, and high level of pathology when abundant Aβ reactive aggregates were noted (Fig. 1).

This grading system was applied on all iNPH samples and on each core in the reference TMA. When the extent of pathology differed between the two cores obtained from the same case in the TMA, the core with highest extent of pathology was chosen.

Additionally, a subset, 37 cases, of iNPH samples, were morphometrically analyzed using the positive pixel count algorithm (version 9.1) within the Aperio ImageScope software (Leica Biosystems, Inc). All settings were pre-set in the software, except “The Intensity Threshold (Upper Limit) of WEAK positive pixels”, which was increased from 220 to 255. The algorithm was applied on grey matter area in the biopsies, excluding the molecular cell layer and vessels with cerebral amyloid angiopathy. The immunoreactivity (IR), with different intensities, was noted in every sample and subdivided into weak, moderate, and strong. The staining quality and the structures visualized by protein expression determined the approach used to quantify the IR pixels, i.e., the extent of pathology. For the Aβ, the sum of all positive pixels were counted independently of the staining intensity. Only the strong- and moderately- positive pixels were counted in the HP*τ* staining. The positive pixels within the grey matter were transformed into a stained area in mm^2^. The ratio between the stained area, per total area in each biopsy ×100, resulted in stained area fraction (SAF), which is a final measure of IR within the tissue.

### Statistical analysis

The statistical analyses were performed using IBM SPSS statistics software, version 27 (IBM Corp, NY, USA). Means and standard error of means (*m*±SE) were used to describe the cohort. Non-parametric tests, Mann Whitney U test (MWU), Kruskal Wallis test (KWT), and Wilcoxon signed-rank test (WSRT) were used to assess differences between groups. The correlations between the studied variables were defined using non-parametric Spearman’s rho two tail test.

## RESULTS

### iNPH subjects

A total of 127 samples from subjects with iNPH were assessed. The age range when the biopsy was taken was 70 to 88 years; moreover, the *m*±SE of age was 77.46±0.43 years, 64 were females and 63 males. The *m*±SE of age for females was 76.98±0.61 and 77.95±0.91 for males. No significant age difference was observed between the genders (MWU, *p* = 0.288). Light microscopic assessment confirmed the presence of grey matter in the biopsies and excluded other processes that could affect the interpretation of the pathology seen as well as confirmed the compartmentalization of the IR assessed with the different IHC markers, i.e., intraneuronal for HP*τ* and extracellular for Aβ. Cerebral amyloid angiopathy was observed in 11 out of the 127 samples, representing 9% of the total cohort.

All cases included displayed Aβ^6F/3D^ positive aggregates, at moderate to high level in their biopsies. HP*τ* was seen in 115 samples (91%) of which 72 subjects (57%) displayed low-, 29 (23%) intermediate-, and 14 (11%) high-level of pathology. To test the assessment strategy applied in this study, the SAF was measured for HP*τ* and Aβ^6F/3D^ in 37 of the iNPH samples.

When assessing HP*τ* pathology, a significant (*p* =0.000) correlation (Sperman’s rho = 0.8) was seen between the outcome applying morphometry (SAF values) and semi-quantitative assessments (low/1, moderate/2 and high/3 level). The *m*±SE of HP*τ*/SAF in low level was 0.28±0.06 % (range 0.02–0.92); moderate level 0.56±0.11 (range 0.32–1.17) and high level 9.18±1.91 (2.91–18.51).

When assessing the Aβ pathology, a significant (*p* = 0.041) correlation (Spearman’s rho = 0.3) was seen between the outcome applying morphometry and semi-quantitative assessment. The *m*±SE of Aβ/SAF in moderate level was 13.81±2.30 (range 7.26–17.00) and in high level, 18.09±0.54 (range 10.00–27.00). The overlap of SAF values in moderate and high level of Aβ is due to the inclusion of the whole biopsy when evaluating the sample by eye contrary to assessing a defined area when applying SAF.

The Aβ^4G8^ positive lesions were detected in all subjects, where 27 (21%) displayed the intermediate level and 100 (79%) displayed the high level of pathology. The umAβ variant was expressed at low level in 7 (6%) of the samples, at intermediate level in 52 (41%) and at high level in 68 (54%) samples. The pyAβ was detected in all samples, wherein 11 (9%) cases at low level, 42 (33%) at intermediate level and 74 (64%) at high level of pathology. The pAβ was not observed in 3 (2%) of the samples, seen at low level in 76 (60%), intermediate level in 46 (36%) and high level in 2 (2%) cases.

The expression of the different proteins studied here was not affected by gender (MWU). The samples were divided into three age groups, depending on the age of the subject when the sample was taken (Table 2). Group 1 included subjects aged 70 to 74 years, group 2 aged 75 to 79, and group 3 aged 80 to 88 years. The IR of the proteins in relation to age groups is summarized in Table 2. A significant difference related to age was observed for three of the proteins, i.e., Aβ^6F/3D^, Aβ^4G8^, and umAβ. In age group 3, the extent of protein expression decreased slightly for all the proteins compared to group 2, but significant decrease was seen only with Aβ^4G8^ (MWU, *p* = 0.017). The decrease of protein expression was absent when only male subjects were included. The decrease was significant (MWU *p* =0.019) for Aβ^4G8^ and close to significant (MWU, *p* = 0.051) for Aβ^6F/3D^ in females. When comparing the mean extent of pyAβ with pAβ, the mean value of pyAβ was higher (45%) for the whole cohort. This difference, pyAβ> pAβ, was also significant (*p* = 0.000, WSRT) in the different age groups.

**Table 2:** The level of protein expression within different age groups. Significance level is 0.05, provided in bold

Age	Age group	Number	Gender	Value	HP*τ*	Aβ				
			F/M			6F/3D	4G8	um7H3D6	*pyN3pE*	*p1E4E11*
70–88	All	127	64/63	m±SE	1.35±0.80	2.71±0.46	2.79±0.41	2.48±0.60	*2.50*±*0.65*	*1.37*±*0.56*
70–74	1	43	23/20	m±SE	1.21±0.11	2.58±0.08	2.67±0.07	2.28±0.10	*2.35*±*0.11*	*1.30*±*0.08*
75–79	2	45	23/22	m±SE	1.51±0.13	2.84±0.06	2.93±0.04	2.62±0.08	*2.60*±*0.09*	*1.40*±*0.08*
80–88	3	39	18/21	m±SE	1.33±0.12	2.69±0.08	2.74±0.07	2.54±0.09	*2.54*±*0.10*	*1.41*±*0.10*
Statistics/KWT					ns	***p* = 0.025**	***p* = 0.009**	***p* = 0.032**	*ns*	*ns*

HP*τ*, hyperphosphorylated *τ*; Aβ, amyloid β; F, females; M, males; um, unmodified; py, pyroglutamylated; p, phosphorylated; *m*±SE, mean±standard error of means; KWT, Kruskal-Wallis Test.

In the total cohort, the extent of all protein altera-tions correlated significantly with each other (Table 3). Within the age groups 2 and 3, where the pathology was pronounced, the HP*τ* pathology correlated at a significant level with both the pyAβ and pAβ but not with Aβ^6F/3D^ and Aβ^4G8^. The umAβ correlated with HP*τ* only in age group 2.

**Table 3:** Spearman’s rho correlations and significance^p^. Correlation is significant at the 0.01 level (2-tailed)

		HP*τ*	Aβ			
			6F/3D	4G8	um7H3D6	pyN3pE
Aβ	6F/3D	**0.33^***0.000***^**
	4G8	**0.28^***0.001***^**	**0.81^***0.000***^**
	um7H3D6	**0.32^***0.000***^**	**0.72^***0.000***^**	**0.58^***0.000***^**
	pyN3pE	**0.41^***0.000***^**	**0.69^***0.000***^**	**0.69^***0.000***^**	**0.63^***0.000***^**
	p1E4E11	**0.34^***0.000***^**	**0.35^***0.000***^**	**0.26^***0.003***^**	**0.42^***0.000***^**	**0.38^***0.000***^**

HP*τ*, hyperphosphorylated *τ*; Aβ, amyloid β; um, unmodified; py, pyroglutamylated; p, phosphorylated.

### Reference material

The TMA included core samples from 19 reference cases, 9 females and 10 males, age range at death 50 to 93 years, *m*±SE 75.74±11.14. The Aβ^4G8^ was seen in all cores, the Aβ^6F/3D^ and the pyAβ in 18 (95%), umAβ in 14 (74%), and pAβ in 11 (58%) of the 19 samples. HP*τ* was seen in all of the samples. Cerebral amyloid angiopathy was observed in 5 (26%) samples.

The reference cases in the TMA were split into three groups, according to the level of ADNC pathology, as recommended by the National Institute on Aging and Alzheimer’s Association’s guidelines, into low, intermediate, and high grade of ADNC [14, 15]. The expression of proteins studied here in samples from subjects with various levels of ADNC is summarized in Table 4. The expression of all studied proteins increased with increasing level of ADNC, and at a significant level for HP*τ*, Aβ^6F/3D^, the umAβ and pAβ variants (KWT). When comparing the mean extent of pyAβ with pAβ, the mean value of pyAβ was 37% higher in the whole TMA cohort as well as in the different levels of ADNC (62%/low level, 50%/intermediate level, 13%/high level). These differences were significant (*p* = 0.002) in the total cohort of 19 samples.

**Table 4:** The level of protein expression within the amygdala samples from subject with different level of Alzheimer’s disease neuropathological change. Significance level is 0.05 is given in bold

Level of ADNC	Number	Value	HP*τ*	Aβ				
				6F/3D	4G8	um7H3D6	*pyN3pE*	*p1E4E11*
All	19	m±SE	2.32±0.89	1.89±0.81	2.42±0.69	1.26±0.99	*1.84*±*0.90*	*1.16*±*1.17*
Low	6	m±SE	1.33±0.82	1,17±0.75	2.17±0.98	0.67±0.82	*1.33*±*1.03*	*0.50*±*0.84*
Intermediate	7	m±SE	2.57±0.54	2.00±0.58	2.43±0.54	1.00±0.82	*1.71*±*0.76*	*0.86*±*1.22*
High	6	m±SE	3.00±0.00	2.50±0.55	2.67±0.52	2.17±0.75	*2.50*±*0.55*	*2.17*±*0.75*
Statistics/KWT			***p* = 0.005**	***p* = 0.015**	ns	***p* = 0.024**	ns	***p* = 0.031**

HP*τ*, hyperphosphorylated *τ*; Aβ, amyloid β; um, unmodified; py, pyroglutamylated; p, phosphorylated; *m*±SE, mean±standard error of means; KWT, Kruskal-Wallis Test.

Within this cohort of 19 samples from amygdala region, HP*τ* correlates with all Aβ variants except for Aβ^4G8^. All the Aβ variants correlate with each other except for Aβ^4G8^, which does not correlate with the umAβ and pAβ variants.

## DISCUSSION

In this study, for the first time, we assessed different biochemical Aβ variants and their association with HP*τ* in a unique cohort of surgical brain biopsies from iNPH subjects undergoing a curative VPS insertion. We chose to include subjects who were 70 years and older with notable Aβ pathology. Our cohort included 127 subjects, all expressing Aβ^6F/3D^ and Aβ^4G8^ at a moderate to high level in line with our selection criteria. Parallel with Aβ, HP*τ* was seen in 91% of these brain biopsies; thus, the cases mirror subjects with different stages of ADNC [25, 26]. Noteworthy, the detection of HP*τ* in 91% of all samples is an outcome that is higher than previously reported by others in the iNPH setting [6, 7, 9, 33]. In this study, the stainings were standardized and automatically performed. We used semi-quantitative grading scheme, and we correlated the outcome of our semi-quantitative grading with morphometric technique. The semi-quantitative grading scheme correlated at significant level with SAF for assessment of both Aβ and HP*τ*, confirming that our method is reproducible and thus reliable.

Regarding the assessment, all labelling, also small grains or thread were assessed, as visualized in Fig. 1A. The assessment strategy of HP*τ* pathology has not been described in detail in previous publications, but while reading the descriptions it seems that only tangles and neurites have been taken into account [6, 9, 10, 33]. In a recent publication by us, it was estimated that when all subjects with sparse HP*τ*/SAF were excluded, i.e., subjects with grains but lacking tangles, the incidence of subjects with HP*τ* is in line with what has previously been reported [8]. Furthermore, contrary to previous publications only cases with notable Aβ pathology were included, i.e., cases with sparse Aβ expression in the cortex were excluded [6, 25, 26]. Moreover, in our cohort, the age ranged from 70 to 88 years, whereas other studies have included subjects from the age of 28 to 87 years. The influence of age on the progression of ADNC is well established [6, 25, 26].

All variants of Aβ studied here increased with age, a finding in line with the known progressive nature of the Aβ process [24, 25, 27, 28, 32]. Different biochemical modifications of Aβ protein have been described, where these modifications have been associated with increased aggregation and neurotoxicity [22, 29]. Both pyAβ and pAβ variants have been detected in Aβ aggregates in mouse models and brain tissue from subjects with pre-clinical AD, AD, and Down syndrome [27, 28, 34]. Previously, it has been reported that aggregation of different biochemical variants of Aβ occurs in a certain hierarchical manner. The pyAβ and pAβ variants are not detected at the early biochemical stage 1. In stage 2, the pyAβ is additionally identified within the aggregates; in the final stage 3, the pAβ variant is detected. The pAβ variant is seen mainly in subjects with symptomatic AD disease [27, 28]. In our cohort, the pyAβ was detected in all samples, while the pAβ was seen in 98%. The high expression of both these modified Aβ variants in our cohort is interesting as it indicates strongly that our cases with notable Aβ aggregation have already surpassed the early stage of pathology, i.e., Thal phase 1 [3]. Thus, this outcome is also in line with our observation that HP*τ* pathology is seen in 91% of the subjects, suggesting that the stage of the neuronal degeneration, i.e., HP*τ* pathology is closer to Braak stages III–IV than Braak stage I–II [4]. It should be noted that we were not able to grade our cohort biochemically regarding Aβ, as has been done previously when studying PM brain with AD [27, 28]. Here based on the selection criteria, we excluded cases with low level of Aβ pathology. Interestingly, the pyAβ expression was significantly higher in all age groups when compared with the pAβ variant (Table 2). This outcome is in line with previous reports based on PM studies on AD subjects, suggesting that pyAβ precedes the aggregation of pAβ [27, 28].

We observed an association between HP*τ* pathology and the modified Aβ variants, pyAβ and pAβ, in our study cohort, particularly regarding groups 2 and 3, where the pathology was more prominent. This is in line with previous reports finding that pyAβ is associated with HP*τ* pathology, particularly in frontal cortical areas. When detected in the frontal cortex, the pyAβ variant has been reported as being a significant predilection marker of AD [30].

We have not assessed the association of the pathology assessed here with the cognitive status of the subjects in our cohort. Previous studies of iNPH subjects have shown that a substantial number of subjects with ADNC in their brain biopsies exhibit cognitive decline at the time of the biopsy and progress into AD during follow-up [7, 17, 18, 36]. Moreover, subjects with cognitive symptoms, but without ADNC in a biopsy, do display ADNC when re-biopsied later [9]. Thus, the presence of ADNC and the pAβ variant in particular, in the brain biopsies of iNPH subjects, certainly suggests that dementia illness associated with ADNC is progressing.

The umAβ^7H3D6^ is an antibody directed toward the non-phosphorylated Aβ variant at Serine residue 8 and is described to be highly specific for Aβ variants without any N-terminal modifications, i.e., pyroglutamylation, phosphorylation or nitration [32]. This umAβ variant increases with age and is correlated with all Aβ variants and HP*τ* within our cohort, as seen in Tables 2 and 3. The umAβ variant, together with pyAβ and pAβ, in the Aβ aggregates, indicates a complex biochemical process during the plaque/aggregate formation in the setting of iNPH, as previously described for AD [22, 27–29, 34]. In iNPH, the presence of ADNC, particularly Aβ, is the strongest predictive factor for the development of AD; consequently, the composition of the Aβ aggregates seen in iNPH subjects is indeed of great interest [18]. Different post-translational changes of Aβ protein alter the biochemical properties of the protein, leading to Aβ variants promoting aggregation, reducing solubility and increasing neurotoxicity. All factors listed above are of significance when designing new treatment strategies [22, 29]. Our results suggest that iNPH patients with varying degrees of ADNC alterations in their brain biopsies would certainly benefit from early contact with memory clinics and eventual intervention with neuroprotective and anti-amyloid pharmaceutical treatments.

Interestingly, the expression of all proteins ass-essed here increased generally with age, independent of gender. Surprisingly, however, a slight decrease, in the expression of all assessed proteins was observed between age groups 2 and 3, as illustrated in Table 2. This decrease was, however, only observed in females and was significant for Aβ^4G8^ and close to significant for Aβ^6F/3D^. The number of subjects was relatively high in both age and gender groups, thus suggesting that this outcome is reliable. Whether the gender related outcome is related to the characteristics of proteins assessed or other secondary alterations seen in the brain of subjects with ADNC such as astrogliosis, inflammation, vascular lesions or medication need to be studied further [31, 37–40].

All the proteins studied in our iNPH cohort have also been studied in a reference TMA with PM brain samples from the amygdala of subjects with various levels of ADNC. In line with previous reports in our PM cohort, an increase of pyAβ and pAβ was observed, parallel with the increase in the level of ADNC; moreover, extent of pyAβ was higher than pAβ at all levels of ADNC (Table 4). These observations are in line with previous reports, where the pyAβ and pAβ were detected in hierarchical manner [27, 28, 34]. Thus, our results from the reference TMA are in line with our observations in the iNPH cohort and confirm previous observations seen in mouse models and human brain tissue from subjects with pre-clinical AD, AD, and Down syndrome [27, 28, 34].

In conclusion, for the first time, we were able to demonstrate modified Aβ variants in brain biopsies from iNPH subjects. Our results indicate that in iNPH, the pyAβ precedes the aggregation of pAβ during the ADNC progression and that the modified variants of Aβ correlate with the HP*τ* in the frontal cortical areas of human brain. These results are congruent with what has been reported for AD [27, 28, 30, 34]. The modified Aβ variants have been reported as being associated with cognitive decline [27, 28]. Our results, and the previously reported association between modified Aβ and cognitive impairment, strongly suggest that iNPH subjects with notable ADNC and cognitive impairment are subjects suffering from AD. Whether the clinical malady caused by ADNC is further altered by pathological lesions in the white matter, related to iNPH, is unclear and should be studied further. Thus, iNPH patients with ADNC in their brain biopsies certainly benefit from contact with memory clinics and treatments that are available modifying ADNC pathology.
